# Chemotherapy-induced zosteriform lichen planus following mastectomy: A rare case report

**DOI:** 10.1016/j.ijscr.2023.109177

**Published:** 2023-12-19

**Authors:** Zuha Shyma, Ankitha Adiga, Myfanwy Joanne D'Souza, Adarsh V.V.

**Affiliations:** a Department of Pharmacy Practice, Karavali College of Pharmacy, Vamanjoor (post), Mangalore 575028, Karnataka, India; b Department of Dermatology, Father Muller Medical College, Mangalore 575028, Karnataka, India

**Keywords:** Dermatology, Adverse drug reaction, Koebner phenomenon, Blaschko's line

## Abstract

**Introduction:**

Zosteriform Lichen Planus represents a relatively uncommon variant of LP. It is characterized by a distinctive distribution following Blaschko's line and involving multiple dermatomes, setting it apart as a unique manifestation. There have been several cases of cutaneous LP reported, but relatively few of them presented as zosteriform LP. To our knowledge, this is the first documented case of zosteriform LP as an adverse drug reaction.

**Presentation of case:**

A 62-year-old female presented to the dermatology clinic with asymptomatic hyperpigmented patches that exhibited gradual spreading. The patient had a history of breast cancer and underwent a mastectomy procedure, chemotherapy, adjuvant therapy, and radiation treatment. A dermatological examination revealed the presence of multiple hyperpigmented, ill-defined macules arranged linearly on the left flank and inner thigh.

**Discussion:**

A biopsy confirmed the diagnosis as Lichen Planus. The patient's condition significantly improved following a nine-week topical steroid with dose tapering.

**Conclusion:**

Zosteriform LP is a rare adverse skin reaction associated with chemotherapeutic drugs. The immunosuppression induced by chemotherapy may trigger T-cell activation, leading to a lichenoid tissue reaction. A thorough patient history assessment is essential for the management of such adverse reactions.

## Highlights

• The distinct feature of zosteriform LP is its distribution, which follows Blaschko's lines
• Drugs can induce LP by acting as haptens that attach to keratinocytes/melanocytes, subsequently triggering a cytotoxic T lymphocyte response
• Lichenoid drug eruptions resolve within weeks to months after discontinuation of the offending drug.
• Sometimes steroids may be required when drug cannot be discontinued

## 1 Introduction

Lichen Planus (LP) is a relatively common papulosquamous skin disorder of undetermined etiology. An unusual variant, known as zosteriform lichen planus, may manifest spontaneously or at the site of a prior herpes zoster or varicella-zoster virus infection. LP is recognized as an immune-mediated disorder affecting both mucosal membranes and the skin. The distinct feature of zosteriform LP is its distribution, which follows Blaschko's lines and encompasses multiple dermatomes, setting it apart from conventional LP [1].

Drug-induced LP, also known as lichenoid drug eruption, is a rare cutaneous adverse effect associated with various medications [2]. The condition presents as a symmetrical eruption of flat-topped, violaceous, or erythematous papules resembling lichen. The latent period between initiating the suspected drug and the onset of cutaneous lesions can range from weeks to over a year, influenced by factors such as drug dosage, host reaction, drug class, and concomitant medications. There have been several cases of cutaneous LP reported, but relatively few of them presented as zosteriform LP. To our knowledge, this is the first documented case of zosteriform LP as an adverse drug reaction [3]. The work has been reported in line with the SCARE criteria [4].

## 2 Presentation of case

**Fig. 1:**
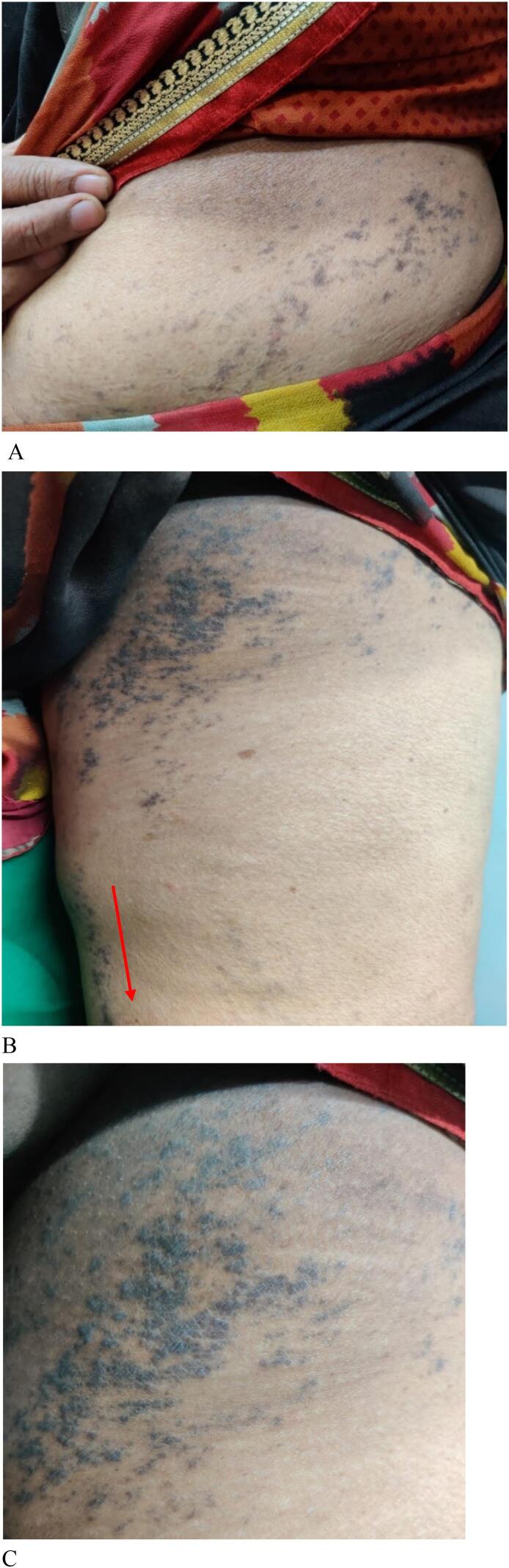
A: Well-defined macules and papules extending from the left infra-mammary area, the lower left side of the abdomen. B: Well-defined macules and papules over the left inner thigh reaching the left mid-calf area. C: lesions showing Blaschko's lines.

A 62-year-old Asian female presented to the dermatology clinic with asymptomatic multiple hyperpigmented patches, which were gradually spreading in nature. The patient had a past medical history of breast cancer and underwent treatment for it. A Trucut biopsy from the left breast was suggestive of infiltrating ductal carcinoma. She underwent left modified radical mastectomy axillary dissection under general anesthesia. She received chemotherapy- 4 cycles of Epirubicin (150 mg) + cyclophosphamide(1100 mg) and 4 cycles of paclitaxel (300 mg), along with adjuvant therapy which included antiemetic, proton pump inhibitor, and systemic steroid. She was started on T. Letrozole 2.5 mg and later received radiotherapy to the left chest wall, supraclavicular and axillary regions. One month after radiotherapy, dermatological examination showed multiple hyperpigmented, well-defined macules and papules in a whorled and linear fashion extending from the left infra-mammary area, the lower left side of the abdomen (*as shown in* Fig. 1) over the left inner thigh reaching the left mid-calf area as shown in Fig. 1B. The lesions occurred spontaneously without any prior herpes zoster or varicella-zoster virus infection.

**Fig. 2:**
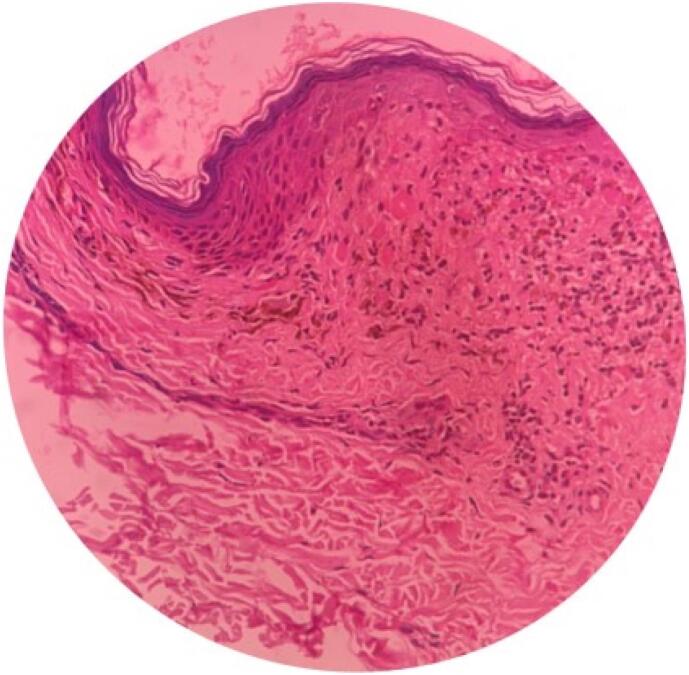
Histopathological appearance of Lichen Planus.

**Fig. 3:**
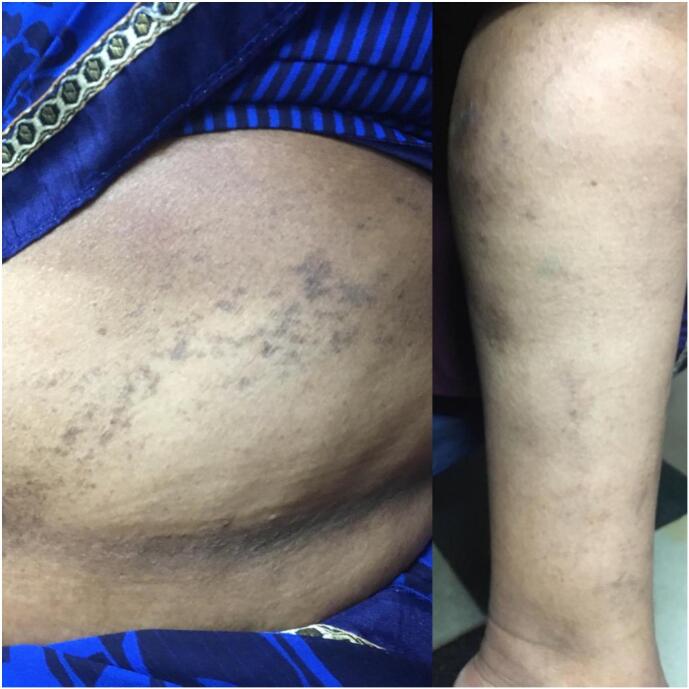
Improvement in patient condition post 9 week follow up.

In order to confirm the diagnosis, an incisional skin biopsy was done from the left inner thigh for a histopathological examination. As shown in Fig. 2, the epidermis showed hyperkeratosis, wedge-shaped hypergranulosis, and vascular alteration of the basal layer, with a moderate degree of lymphocytic infiltrate seen at the dermo-epidermal junction with pigment incontinence confirming Lichen Planus. The distribution of the lesion is multi-dermatomal (T 11, T 12, L1-L3). It was found to follow Blaschko's lines (Fig. 1C), and so she was diagnosed with Zosteriform Lichen Planus induced by chemotherapy. Causality assessment indicated a “possible” relationship, with a score of 4 on the WHO's (World Health Organization) Naranjo Causality assessment scale. The development of zosteriform lichen planus is plausible due to chemotherapy-induced immunosuppression. The patient was prescribed a nine-week regimen of topical steroids (betamethasone 0.05 % + zinc sulfate 0.5 %). Subsequently, the patient's condition exhibited marked improvement (Fig. 3).

## 3 Discussion

Lichen Planus (LP) remains a common papulosquamous skin condition with an unknown etiology. Zosteriform LP, characterized by a dermatomal arrangement and adherence to Blaschko's lines, represents an unusual variant and may be explained as Köebner phenomenon [5]. Such cases may be underreported due to the variable latent period between drug initiation and the appearance of cutaneous lesions. Thoroughly reviewing medication use over the preceding 12 to 14 months is imperative in diagnosing drug-induced lichen planus [6]. Drugs can induce lichen planus by acting as haptens that attach to keratinocytes or melanocytes, subsequently triggering a cytotoxic T lymphocyte response [7].

Lichen Planus is generally regarded as an idiopathic disease. The prevailing theory suggests that contact with exogenous substances, such as viruses, medications, or allergens, modifies epidermal self-antigens and activates cytotoxic CD8+ T cells. This interaction between altered and normal self-antigens on basal keratinocytes leads to T-cell targeting and apoptosis. In the context of chemotherapy-induced immunosuppression, T-cell activation is likely to have played a role in triggering the lichenoid tissue reaction. Even though Chemotherapy-induced hyperpigmentation is widespread regardless of race, it is more frequent and prominently seen among dark-skinned individuals [8]. The identification of the specific drug or drugs responsible for adverse skin reactions can be challenging, particularly in cases involving multiple drugs, as seen in this case [9].

Since radiation was specifically administered in chest region, occurrence of lesion beyond chest region nullifies the relation between radiation therapy and zosteriform lichen planus.

Lichenoid drug eruptions typically resolve within weeks to months after discontinuation of the offending drug. Symptomatic patients with limited skin area involvement are recommended to receive topical corticosteroids, while oral corticosteroids are advised for extensive, symptomatic disease. In cases where systemic corticosteroids are contraindicated, oral retinoids may serve as an alternative.

Healthcare providers should be vigilant, as drug use may underlie certain LP cases, and consultation with treating physicians is essential [10]. Withdrawal of the suspected drug leading to the gradual disappearance of lesions serves as confirmation of the diagnosis. Previous reports have detailed Pembrolizumab-Induced Lichen Planus and Lichenoid reactions induced by Adalimumab [11,12]. However, this case represents, to our knowledge, the first documented case of zosteriform lichen planus induced by chemotherapy.

## 4 Conclusion

Zosteriform lichen planus is a potential adverse skin reaction associated with chemotherapeutic drugs. Thorough patient history assessment and vigilance in monitoring for such reactions are crucial for timely diagnosis and management.

## Consent form

Written informed consent was obtained from the patient for publication of this case report and any accompanying images. A copy of the written consent is available with the corresponding for review by the Editor-in-Chief of this journal upon request.

## Provenance and peer review

Not commissioned, externally peer reviewed.

## Ethical approval

The Father Muller Institutional Ethics Committee (FMIEC) of Father Muller Research Centre, Kankanady, Mangalore, Karnataka, India, approved the case report for publication on 14th September 2023 with approval number FMIEC/CCM/497/2023.

## Funding

This research did not receive any specific grant from funding agencies in the public, commercial, or not-for-profit sectors.

## Author contribution

**Zuha Shyma** - Writing - review & editing, Writing - original draft, Conceptualization, Investigation.

**Ankitha Adiga** - Conceptualization, Investigation, Writing - review & editing.

**Myfanwy Joanne D'Souza** - Conceptualization, Investigation, Writing - review & editing.

**Adarsh VV** - Writing - review & editing.

## Guarantor

Zuha Shyma.

## Research registration number

N/A.

## Conflict of interest statement

The authors declare that they have no conflict of interest.
